# Polish Firefighters’ Participation in Interventions Related to Behavioral Disorders in the 2020–2022 Period: An Observation of Incidents

**DOI:** 10.3390/healthcare12232482

**Published:** 2024-12-09

**Authors:** Lukasz Dudzinski, Lukasz Czyzewski, Robert Gałazkowski, Filip Jaskiewicz, Klaudiusz Nadolny, Tomasz Kubiak

**Affiliations:** 1 Department of Medical Rescue, John Paul II University in Biala Podlaska, Sidorska Str. 95/97, 21-500 Biała Podlaska, Poland; lukasz_dudzinski@o2.pl; 2 Department of Geriatric Nursing, Warsaw Medical University, Zwirki i Wigury Str. 61, 02-091 Warsaw, Poland; lukasz.czyzewski@wum.edu.pl; 3 Department of Emergency Medical Services, Warsaw Medical University, Zwirki i Wigury Str. 61, 02-091 Warsaw, Poland; robert.galazkowski@wum.edu.pl; 4 Emergency Medicine and Disaster Medicine Department, Medical University of Lodz, 90-419 Lodz, Poland; 5 Department of Emergency Medical Service, Faculty of Medicine, Silesian Academy in Katowice, 40-555 Katowice, Poland; prrm.knadolny@interia.pl; 6 Department of Health Sciences, Poznan Medical Academy of Applied Sciences Mieszko I, Poznan, Bulgarska 55, 60-320 Poznan, Poland; tomaszkubiak@tom-rat.eu

**Keywords:** rescue and firefighting operations, support for medical units, mental disorders, suicide attempt, health risks

## Abstract

Background: Rescue service interventions for patients with behavioral disorders are quite common. The aim of this study is to analyze interventions of Polish State Fire Service units in incidents involving health threats to victims with behavioral disorders. Materials and Methods: This study used data from the Decision Support System of the State Fire Service. Events in the period of 1 January 2020 at 00.00–31 December 2022 at 23:59 were used for analysis. Results: In the 2020–2022 period, firefighters were dispatched 321 times to interventions concerning injured individuals with behavioral disorders. Isolated emergency medical incidents most often concerned mental disorders (23%) and least often concerned suicide attempts (8%) (*p* = 0.033). It was shown that the duration of the intervention (emergency care time) was significantly longer in the case of interventions related to the autumn period (*p* < 0.001). Conclusions: Our results describe parameters associated with behavioral health responses within the Polish fire service.

## 1. Introduction

The Polish National Rescue and Firefighting System (NRFS) has established a structure of cooperation, which includes the forces and resources of the State Fire Service (SFS) and Volunteer Fire Brigades (VFB). This structure is maintained using the state budget and local government budgets. To a firefighter, the most important goal is to protect and save the lives and health of citizens as well as to protect the environment and forecast, identify, and combat threats [1]. 

Firefighters’ emergency response interventions are classified according to two main types of hazards: fires and local threats (LTs). To combat and neutralize the effects of certain hazards, Specialist Rescue Groups (SRGs) (e.g., water and diving rescue, medical rescue, search and rescue, technical rescue, and high-altitude rescue) are used [2,3]. Firefighters provide assistance in the scope of medical rescue at the level of qualified first aid (QFA). Specifically, firefighters participate in the following medical activities:

- Direct activities: These involve medical rescue operations (MROs), such as assistance given to medical units, or isolated emergency medical incidents (IEMIs), when firefighters are the first to intervene at the scene of the incident, and leading units—State Emergency Medical Services (SEMS)—are dispatched but not yet available on the spot.
- Indirect activities: These involve providing assistance to SEMS units other than medical assistance (auxiliary activities or the use of specialized equipment) in order to eliminate health hazards. These are all firefighters’ necessary activities for the effective rescue of an injured person in which firefighters do not directly implement medical procedures [4,5,6].

Interventions by emergency services for patients with behavioral disorders (stress responses, family conflicts, substance abuse, aggravation of symptoms of mental illness, and patients not diagnosed as mentally ill) are quite common. SEMS units are statutorily obliged to undertake these interventions. In many cases, cooperation with other services like the police, forest services, coastal and mountain rescue services, and fire protection units is crucial for the effective completion of operations. Local social care and family are often helpful in these types of incidents [7,8,9]. For most situations that are hazardous to society, a lead service and cooperating entities are assigned. Firefighters carry out many specialized procedures and have a lot of equipment. Some of the activities are carried out by Specialized Rescue Groups (SRGs), whose characteristics in Poland are presented in Table 1.

In areas where the State Fire Service is not a leading service, firefighters closely cooperate with leading entities, e.g., SEMS, the police, the army, the border guard, the sanitary inspection service, the environmental protection inspectorate, the National Atomic Energy Agency, and many others [11].

Firefighters’ rescue operations are based on QFA procedures. These involve a set of step-by-step rules for dealing with a specific health issue. These include procedures connected to sudden cardiac arrest management, trauma management, and other non-traumatic health problems. An important part of these procedures concerns psychological support. Among others, firefighters are required to establish verbal contact with the injured person, support patients to become aware of the situation, and inform them about planned decisions and actions (e.g., transferring the patient to a spinal board, immobilizing the neck with a cervical collar, or cutting the vehicle with mechanical or hydraulic devices) [12].

During rescue operations, the procedures for cooperation between services, competencies, and supervision principles should be and are regulated in Poland. For logistic reasons and due to the availability of individual services, however, there are situations in which the service dedicated to a given problem experiences a delay in relation to fire brigades. This leads to the need to take actions that are challenging and beyond the standard preparation of firefighters as part of QFA training programs. These may include mental disorders and behavioral disorders of various etiologies. 

The main goal of this study was to analyze the intervention (the support of medical entities) of firefighters for victims with behavioral disorders. The identification of the needs of the rescue system and the cooperation of firefighters may affect the safety and health of patients and emergency services personnel [13].

## 2. Materials and Methods

### 2.1. Study Design

The material used for analysis was made available by the Operational Planning Office (OPO) with the consent of the Chief Commander of the State Fire Service (HQ of the State Fire Service). Approval was obtained in May 2023. The observations cover the 2020–2022 period on the territory of Poland. This study used data from the Decision Support System of the State Fire Service (DDS SFS) in which all firefighters’ interventions are archived. The quantitative analysis was fully anonymous for all people linked with the described events (addresses, dates of events, service personnel, and injured individuals); therefore, no opinion was sought from the bioethics committee. We received the data in MS Excel files.

### 2.2. Statistical Analysis and Search Processing

Results for quantitative variables are presented as mean values ± standard deviations. A one-way analysis of variance (ANOVA) was used to characterize the comparative intervention time. Qualitative variables were presented as quantitative values (n) and percentages of the total group (%), while proportions within groups were assessed using the chi-square test. Statistica 13 software (StatSoft Inc., Tulsa, OK, USA) was used for statistical analysis. The significance level was *p* < 0.05.

The inclusion criteria for the analysis are as follows:

1. Events in which fire brigade units intervened to help an injured person with a mental disorder regardless of the coding of the event (fire/LH).
2. Direct and auxiliary actions of firefighters in incidents with the medical problem in question being considered.
3. Events within the following range of dates covered by the observation: 1 January 2020 (00:00)–31 December 2022 (23:59).
4. Interventions carried out as part of rescue and firefighting operations in Poland—including all voivodeships (international operations were not considered).
5. Searching for the following keywords in DSS-SFS in the categories of IEMI and MRO as the reasons for the call: “mental disorders”, “suicidal disorders”, “suicidal thoughts”, “S thoughts”, “self-harm”, “behavioral disorders”, and “mental illness”. The authors focused on behavioral disorders without analyzing whether a given inured individual had been diagnosed with a mental illness. Our analysis included people whose behavior posed a threat to themselves or the environment and could have been a one-off episode. The described interventions include individual cases of diseases other than mental illnesses that affected the most important cognitive functions of a person, namely memory, logical thinking, and orientation regarding the place and time in which a given individual is. The documentation described neurodegenerative diseases (Alzheimer’s), senile diseases (dementia), the effects of psychoactive substances (mainly ethyl alcohol), metabolic diseases (diabetes), autism, and Asperger’s. Our data are supplemented by data from injured individuals with behavioral disorders after a head injury or following a traffic incident.
6. The use of drones to search for people with mental disorders.

## 3. Results

After considering the inclusion criteria and keywords used in the search of the databases of the State Fire Service in the 2020–2022 period, firefighters were dispatched 321 times for operations related to individuals’ behavioral disorders (286 IEMIs + MROs and 35 drone searches). In addition, firefighters provided psychological support during MROs. Table 2 describes the general characteristics of the population and firefighters’ interventions in the years covered by the observation while considering the procedure of psychological support. The sample size has a 95% confidence level and a maximum error of 6%.

Table 3, Table 4, Table 5 and Table 6 include statistical calculations of the data included in the analysis using variables that could be generated from the DSS-SFS database: the total number of interventions, the intervention duration, the daily time of intervention, the subtype of intervention, and the reason for the call. As part of the study, a comparative analysis was conducted on the impact of the time periods (2020 vs. 2021 vs. 2022) in which interventions in connection with mental/behavioral disorders were performed on the factors associated with their occurrence (Table 3). There was a significant relationship between the analyzed period and intervention type (*p* = 0.023). No significant differences were found for the intervention period (day vs. night; *p* = 0.345) or the subtype of incident (isolated emergency medical incidents vs. LT-SEMS assistance; *p* = 0.583). The different types of interventions carried out during the observed period had no impact on the time of day or on the implementation of the interventions.

In the conducted study, the influence of factors related to the characteristics of rescue operations (IEME and LT-SEMS assistance) and intervention time [min] was analyzed (Table 4). It was shown that the duration of the intervention was significantly longer in the case of interventions related to the autumn period (*p* < 0.001). It was shown that the intervention time was significantly longer in the case of psychiatric disorders (*p* = 0.003). The type of incident (*p* = 0.073) and the time of intervention (*p* = 0.182) were not related to the observed intervention time.

In this study, the impact of factors related to the use of drones with the intervention time [min] was analyzed (Table 5). It is shown that the intervention time was significantly longer when searching for women (*p* = 0.036). It was shown that the intervention time was significantly longer when interventions were conducted between 7.00 a.m. and 7.00 p.m. (*p* = 0.017) and during searches using dogs (*p* < 0.030). The type of intervention (*p* = 0.792) and age had no significant effect on the observed intervention time. The age of wanted persons was not associated with the intervention duration (*p* = 0.057).

In some of the incidents in which a drone was dispatched, when the time criterion was an important factor and the area to be searched was significant, there were additionally dispatched groups with rescue dogs operating in the State Fire Service, dedicated mainly to conducting international operations in construction disasters. However, with the availability of a search and rescue group, the Rescue Operations Officer (ROO) may decide on becoming involved in local operations. 

A comparative analysis of the causes of interventions showed that IEMIs most often concerned mental disorders (23%), and suicide attempts were least often involved (8%) (*p* = 0.033). No statistically significant effect of the time of intervention (*p* = 0.473) and season (*p* = 0.338) on the cause of intervention was found (Table 6). 

Age and gender data were not included in many intervention descriptions (reports). In the documentation of the State Fire Service, the following description was often given: “injured person or wanted person”. This is explained in detail in the section titled “Limitations and Future Prospects”; the descriptive content of the information from the incident depended on the Rescue Operations Officer (ROO), which the authors had no influence on. 

## 4. Discussion

To the authors’ knowledge, there are few studies available on the use of firefighters’ equipment and procedures to rescue people with mental disorders. Our data indicate that medical units provide this type of support quite frequently, and there may be an upward trend. 

Apart from the results concerning the main purpose of our observation, it is worth mentioning the QFA procedure concerning psychological support, which is realized during each intervention with conscious injured persons. Thanks to the comprehensive care of firefighters, many injured people generate less situational stress related to a given incident. Contact with an injured person during operation and providing accurate information to an injured person help them become oriented in the situation and in their location and help to reduce the stress reaction to a given incident. Firefighters provide psychological comfort to injured individuals through the additional steps of this procedure: moving bystanders away, ensuring intimacy (shielding and covering), maintaining a close presence to the injured individual throughout the operation, using a calm tone of voice, and introducing their name, surname, and rank; all of these are carried out to inspire trust in the injured person [15,16]. 

UAVs are a technology widely used in many fields due to their ability to explore a large area of land in a short period of time without involving a significant number of people. A drone equipped with cameras and a thermal imaging option is able to penetrate a large area that is difficult for people to access on foot (mountainous regions, swamps, wetlands, and river backwaters) [17,18]. A univariate analysis conducted in the present study showed that in all 35 cases when drones were used, the intervention time was significantly longer for female patients (x = 417 ± 207 vs. x = 258 ± 133; *p* = 0.036), for operations carried out between 7.00 a.m. and 7.00 p.m. (x = 320 ± 151 vs. x = 197 ± 104; *p* = 0.017), and for operations demanding the use of dogs (x = 389 ± 197 vs. x = 255 ± 124; *p* < 0.030). While the reasons for this may seem unclear based on gender, it is surprising that the time of intervention in relation to daylight hours and the use of additional resources in the form of search dogs were associated with extended intervention times. Due to the small number of cases, these results may be difficult to generalize but should draw attention from the perspective of future research.

A significant proportion of the incidents included in the analysis were suicide attempts/thoughts, and there was a significant relationship between the analyzed period and intervention type (*p* = 0.023). From the perspective of both public health and the challenges faced by services aiming to help patients, the increase in the number of suicide attempts in which firefighters intervened or provided support may be particularly worrying. Numerous studies indicate that this is a growing phenomenon. With the growing interest in the phenomenon of suicide in recent decades, more and more information about interventions and social education is emerging. Obtaining a fuller understanding of self-destruction along with helping people and their loved ones in suicidal crises can help reduce the number of sudden deaths. Effective help in this type of mental health crisis should include practical psychological support for people in psychologically difficult situations and help provided in the spirit of humanistic respect for personal dignity, cultural differences, and social diversity [19,20]. 

According to Kawecki, suicide, which is a manifestation of social and personality disintegration, is a social phenomenon of interest to many scientists of various disciplines. In his analysis of the 2008–2018 period on the territory of Poland, the author demonstrated two trends: an increase in the total number of suicide attempts (2008—n = 5237; 2018—n = 11,167) and a decrease in deaths as a result of these attempts, expressed as a decrease from 75% in 2008 to 46% in 2018 [21]. In the present analysis, Polish firefighters intervened in 106 cases categorized as being related to mental disorders and as many as 119 cases of suicidal thoughts and 61 suicidal attempts. In the years covered by the analysis, the latter also show a slight upward trend, although a full interpretation is still not possible due to the relatively small number of cases in individual years; this issue should be monitored and analyzed annually.

The downward trend in deaths from suicide attempts may be related to victims who want to attract attention, to the development of psychological help in specialist centers, to the development of therapy, and, to some extent, or as our results indicate, to the better equipment available in emergency services, dispatch procedures, procedures to be followed during interventions, and the cooperation of several units in one intervention.

The cooperation of services in cases of intervention for behavioral disorders is very important. A 2023 study on the intervention of Emergency Medical Teams (EMTs) for patients with mental disorders showed EMT cooperation with the police in 40% of cases. The police are the leading service in the case of people posing a threat to themselves, other people, or the staff of medical services. In our analysis, we found that the police also often took part in interventions, and in the case of searching for a sick person with a drone, they were the leading service. We have not compared police interventions in our activities [22].

Tymiński draws attention to a different type of cooperation between SEMS and firefighters in terms of medical procedures. Firefighters are often the first to be at the place of call, but an analysis of the competence of QFA rescuers has shown that they are not optimally prepared to assist medical personnel in carrying out emergency medical procedures. This is mainly due to differences in the standard of equipment and education. Firefighters do not have medicine and diagnostic equipment, and only some officers in the firefighter population have medical education. Therefore, firefighters often perform an auxiliary function or provide psychological support in operations for an injured person or a person with behavioral disorders according to the data confirmed in our analysis [23].

The aim of the Prasetyo study was to identify the determinants of mental health disorders in society during the COVID-19 pandemic. The results of the study show that younger people are three times more likely to suffer from mental disorders than older people. In this study, age was not statistically significant, although a clear trend was visible. In our analysis, the exact ages of the injured individuals were not available, but the course of rescue operations shows the entire age spectrum (Asperger’s, mainly seen teenagers, with a predominance of people in many professional activities, and the elderly, who mainly have dementia disorders) [24].

Similar observations were found in another study. Duncan et al.’s review examines the epidemiology of processes and outcomes for individuals for whom an ambulance was called due to a mental health emergency or self-harm. Interventions that were objective based on the analysis accounted for 11% of the total. Some patients were transported to a hospital. It is interesting that within a year of the first intervention (4%), patients often died by suicide [25]. 

Fleury et al. investigated the reasons for calls to patients with mental disorders. Patients under observation were often transported to the ED instead of to a specialist ward, and the profile of the statistical patient was a single person with a low socio-economic status with predisposing factors: suicidal thoughts/attempts, depression, and anxiety [26].

Firefighters have great opportunities to use resources, rescue techniques, and equipment to eliminate the negative effects of this type of threat. Among the articles found that met our assumptions, people who received an intervention had various mental disorders. Jasani conducted a study (2023) on the intervention of firefighters in situations of terrorism (the attackers do not have to be mentally ill, but the behavior of these people is altered and inadequate for the social behavior model). The Global Terrorism Database identified a total of 42 attacks involving firefighters, resulting in 26 deaths and 95 injuries. Of the 42 attacks, 12 (28.6%) were secondary attacks that targeted the firefighters themselves reacting to the first attack [26]. Due to the relatively small number of recorded cases, the results of this analysis should not be generalized; however, the relationship between firefighters and patients reporting suicidal thoughts or, furthermore, patients making suicide attempts, is an undeniable fact. This should draw the attention of scientists and decision makers and contribute to obtaining a better understanding of this public health problem and finding effective solutions to facilitate the activities of firefighters in mental disorder interventions.

In the literature on this subject, there are items describing firefighters with the term first responder. This applies to situations when firefighters are dispatched to a life-threatening situation due to a shorter travel time than an ambulance or due to the lack of availability of an ambulance in a given operational area. No observations were found that directly related to our purpose of work, but the analyses have a common denominator with our results: firefighters cooperate with other services in the rescue system or they are the first to arrive at the scene of the incident.

Svensson et al. described an earlier response by using firefighters as medical first responders while waiting for the ambulance [27]. Berglund et al. pointed out that time is the crucial factor in the “chain of survival” treatment concept, and firefighters’ arrival time is often shorter than that of emergency units [28]. So far, this issue has been understood to refer to the management of trauma and cardiac arrest, but it is worth paying attention to other interventions that are associated with a growing risk to public health—such as mental disorders.

## 5. Limitations and Future Prospects

Our observations have the following limitations:

1. A small proportion of incidents may not have been effectively searched for and included in the analysis due to incorrect classifications by the system or incorrect descriptions by the persons preparing the documentation. The person who prepares a report from an incident is the Rescue Operations Officer (ROO), who, in most cases, does not have medical education, so they do not use medical terminology or use other expressions instead.
2. The incidents covered by the observation period account for a small part of the total number of firefighters’ annual interventions.
3. The ages and sexes of the participating individuals, as well as clinical details, are not available in SFS reports. This analysis is quantitative and spatial in its nature and intends to draw attention to the versatility of firefighters’ actions apart from their basic statutory tasks.
4. Clinical details of the events (basic vital signs and medical diagnoses according to the ICD-10 classification) were not available in the descriptive part of the State Fire Service reports because the intervening SEMS units are responsible for medical documentation. In the documents, there were some ICD-10 codes, e.g., F10 (mental and behavioral disorders caused by alcohol use) and F03 (“unspecified dementia”). The authors did not include ICD-10 codes in the analysis because they appeared in the documents individually [29].
5. The analysis did not include actions in which jump cushions from fire trucks were used to assist people threatening to commit suicide (in approximately 50 interventions per year). The authors did not have access to these incidents; however, this highlights that the share of firefighters dealing with people with mental disorders is higher than presented in our analysis. Assessing this type of incident may be the goal of future analyses.
6. There is no specific definition of mental disorders in the firefighters’ dispatch system. Some of the interventions selected using keywords could be inaccurate, and behavioral disorders could have been revealed only at the stage of assessment conducted by medical teams, which was not included in the firefighters’ documentation. An example is a call for firefighters to forcibly open an apartment at the request of the police or Emergency Medical Team (EMT) with the use of mechanical, hydraulic, or demolition equipment. In this type of call, firefighters end the intervention after completing their task and do not have detailed knowledge of what type of health threat was the cause of the intervention. There are several reasons for this type of intervention: an elderly person with limited mobility, a person with behavioral disorders, a lack of contact from a resident of the premises, a carbon monoxide (CO) detector activated, and suspected criminal reasons, such as persons wanted by the police.

Firefighters’ participation in interventions for victims due to behavioral disorders may be more frequent due to stress, lifestyle, or abuse of psychoactive substances progressing in various populations. SEMS remains a leading service in the field of medical rescue intervention. However, due to the location of their units in Poland, firefighters are often the first responders at the scene. Therefore, there is a need to distinguish mental support activities in the rescue procedures of firefighters. The rescue procedure describes how the lifeguard should behave when contacting the victim in a mental crisis, including the correct tone of voice and attitude to inspire trust. Future research should focus on identifying the needs in the field of reporting, including the assessment of effectiveness and active actions to improve the quality of mental support and constructive responses to stressful situations.

Based on the results obtained and the discussion, the authors defined the strengths and weaknesses of this study. There are several significant advantages of the study that enhance its scientific value and practical importance. On the other hand, the analysis encountered several limitations (Table 7).

## 6. Conclusions

The use of specialized equipment from National Rescue and Firefighting System units provides various opportunities to support emergency medical service units. Investment in new technologies for rescue contributes to the effectiveness of operations. The State Fire Service is the main service cooperating with the State Emergency Medical (SEM) system during medical rescue operations. Psychological support is one of the most commonly used procedures as part of Medical Rescue Operations.

Our results show that behavioral disorders can have many causes, such as disorders experienced in the course of other diseases or disorders caused by a single incident related to a stressful event. Published research related to the aim of this article indicates a lack of in-depth analyses concerning single-country or international perspectives regarding the involvement of firefighters with patients with behavioral disorders.

## Figures and Tables

**Table 1:** Specialist Rescue Groups in the National Rescue and Firefighting System.

Type	Number of Groups
Chemical and Ecological	49
Technical	26
High-Altitude	32
Water and Diving	51
Search and Rescue	21
Other Groups	15
Total	194

Source: www.gov.pl/monitoring-gotowosci-operacyjnej-sgr (accessed on 19 June 2024) [10] [status 31 December 2023].

**Table 2:** The implementation of emergency medical operations, including mental support, in the 2020–2022 period (the data in columns are expressed in thousands).

Year	* Employment	Total MRO	MRO in LT	MRO in Fires	Mental Support
2020	29.9	173.6	158.7	14.9	27.8
2021	30.2	219.0	204.3	14.7	35.0
2022	30.5	187.5	171.1	16.4	30.8

Abbreviations: MRO—medical rescue operations; LT—local threats. * Employment—the number of officers throughout Poland at the end of the observation period. Source: the authors’ own elaboration based on collected data and the Information Bulletin of the State Fire Service for the 2020–2022 period [14].

**Table 3:** Univariate comparison of observation years.

Year	2020	2021	2022	*p*
N	52	131	103	
**Intervention period, N (%)**				
7.00–19.00	34 (65)	92 (70)	63 (61)	0.345
19.00–7.00	18 (35)	39 (30)	40 (39)	
**Cause, N (%)**				
Mental disorders	13 (25)	59 (45)	34 (33)	0.023
Suicidal disorders	22 (42)	53 (41)	44 (43)	
Suicide attempts	17 (33)	19 (14)	25 (24)	
**Subtype of event** **, N (%)**				
LT-SEMS assistance	44 (85)	103 (79)	85 (83)	0.583
IEMI	8 (15)	28 (21)	18 (18)	

Abbreviations: LT—local threat; SEMS—State Emergency Medical Services; IEMI—isolated emergency medical incident.

**Table 4:** Univariate comparison across levels of independent variables: duration of intervention.

	Interventions	Intervention Time [Min]	*p*
Independent Variable Levels	263	18 ± 13	
**Season**			
Spring	61	15 ± 11	<0.001
Summer	45	14 ± 10	
Autumn	96	22 ± 16	
Winter	61	15 ± 10	
**Year**			
2020	45	16 ± 12	<0.001
2021	126	21 ± 15	
2022	92	14 ± 9	
**Intervention period**			
7.00–19.00	174	18 ± 13	0.182
19.00–7.00	89	16 ± 12	
**Cause**			
Mental disorders	100	21 ± 15	0.003
Suicidal disorders	106	15 ± 12	
Suicide attempts	57	15 ± 12	
**Subtype of event**			
LT-SEMS assistance	209	18 ± 14	0.073
IEMI	54	15 ± 9	

Abbreviations: LT—local threat; SEMS—State Emergency Medical Services; IEMI—isolated emergency medical incident.

**Table 5:** Univariate comparison of intervention time (duration) in cases using drones.

	N	Intervention Time [min]	*p*
Total	35	282 ± 148	
**Intervention result**			
Found	3	275 ± 235	0.577
Not found	32	281 ± 149	
**Intervention period**			
7.00–19.00	24	320 ± 151	0.017
19.00–7.00	11	197 ± 104	
**Intervention type**			
Suicide attempt	18	280 ± 131	0.792
Missing/mental disorder	17	284 ± 168	
**Rescue dogs**			
Yes	7	389 ± 197	0.030
No	28	255 ± 124	
**Age (years)**			
<46	11	206 ± 150	0.057
≥46	8	354 ± 182	
**Gender**			
Men	24	258 ± 133	0.036
Women	5	417 ± 207	

**Table 6:** A comparison of the proportion of emergency responders by type.

Reason	Mental Disorders	Suicidal Disorders	Suicide Attempts	*p*
N	106	119	61	
**Intervention period, N (%)**				0.473
7.00–19.00	72 (68)	74 (62)	43 (71)	0.473
19.00–7.00	34 (32)	45 (38)	18 (29)	
**Season, N (%)**				0.338
Spring	20 (19)	26 (22)	18 (29)	0.338
Summer	18 (17)	24 (20)	14 (23)	
Autumn	43 (41)	45 (38)	14 (23)	
Winter	25 (24)	24 (20)	15 (25)	
**Subtype of event** **, N (%)**				0.003
LT-SEMS assistance	82 (77)	94 (79)	56 (92)	0.033
IEMI	24 (23)	25 (21)	5 (8)	

Abbreviations: LT—local threat; SEMS—State Emergency Medical Services; IEMI—isolated emergency medical incident. The *p*-values reflect differences within levels between response types (mental disorders vs. suicidal disorders vs. suicide attempts).

**Table 7:** Strengths and weaknesses of this study.

Strengths	Weaknesses
1. An innovative research design is an important advantage because it allows us to assess the support that firefighters provide to the emergency medical system in difficult interventions for mental disorders. In these interventions, the patient’s behavior is often unpredictable, and firefighters have specialized equipment that is not available in ambulances. Greater forces and resources available for this type of intervention may affect the safety of the patient and the service staff.	1. No data were obtained from the SEMS regarding the availability of ambulances for the described events, including travel times, the length of the intervention, and the completion of the intervention (patient transfer to a hospital, pharmacology, and vital parameters). These data could influence the interpretation and application of the results.
2. This analysis may influence the systemic training process of firefighters in terms of the risks to firefighters’ health that are possible when in contact with victims with mental behavioral disorders or with injured people.	2. The official documentation of the State Fire Service presents little medical information about the injured party; the authors are aware that a significant part of interesting clinical information remains in SEMS documents.
3. The small amount of available studies in the literature that are relevant to our topic shows that this research problem needs to be explored further. Cooperation between services is needed to eliminate many threats in society, and this type of intervention may have an increasing trend.	3. There is a lack of data and tools to learn the reasons for the intervention. Firefighters receive a report of secondary causes, or the “support of medical teams”. However, there is no primary information about what influenced the injured party to seek an intervention (intensification of mental illness, reaction to stress, or psychoactive substances).

Abbreviations: SEMS—State Emergency Medical Services.

## Data Availability

The data presented in this study are available upon request from the corresponding author due to the state’s legal regulations regarding sharing data on internal security relating to country defense or security in crisis activities and units performing the above tasks.
